# Molecular Pathway to Protection From Age-Dependent Photoreceptor Degeneration in *Mef2* Deficiency

**DOI:** 10.1167/iovs.17-21767

**Published:** 2017-07

**Authors:** Saumya Nagar, Dorit Trudler, Scott R. McKercher, Juan Piña-Crespo, Nobuki Nakanishi, Shu-Ichi Okamoto, Stuart A. Lipton

**Affiliations:** 1 Neuroscience and Aging Research Center and Graduate School of Biomedical Sciences, Sanford Burnham Prebys Medical Discovery Institute, La Jolla, California, United States; 2 Neurodegenerative Disease Center, Scintillon Institute, San Diego, California, United States; 3 Department of Neurosciences, University of California, San Diego, School of Medicine, La Jolla, California, United States; 4 Department of Molecular Medicine, The Scripps Research Institute, La Jolla, California, United States

**Keywords:** MEF2D, PGC1α, retinal explant, photoreceptor degeneration, neuroprotection

## Abstract

**Purpose:**

Photoreceptor degeneration in the retina is a major cause of blindness in humans. Elucidating mechanisms of degenerative and neuroprotective pathways in photoreceptors should afford identification and development of therapeutic strategies.

**Methods:**

We used mouse genetic models and improved methods for retinal explant cultures. Retinas were enucleated from *Mef2d^+/+^* and *Mef2d*^−^*^/^*^−^ mice, stained for MEF2 proteins and outer nuclear layer thickness, and assayed for apoptotic cells. Chromatin immunoprecipitation (ChIP) assays revealed MEF2 binding, and RT-qPCR showed levels of transcription factors. We used AAV2 and electroporation to express genes in retinal explants and electroretinograms to assess photoreceptor functionality.

**Results:**

We identify a prosurvival MEF2D-PGC1α pathway that plays a neuroprotective role in photoreceptors. We demonstrate that *Mef2d*^−^*^/^*^−^ mouse retinas manifest decreased expression of PGC1α and increased photoreceptor cell loss, resulting in the absence of light responses. Molecular repletion of PGC1α protects *Mef2d*^−^*^/^*^−^ photoreceptors and preserves light responsivity.

**Conclusions:**

These results suggest that the MEF2-PGC1α cascade may represent a new therapeutic target for drugs designed to protect photoreceptors from developmental- and age-dependent loss.

Dysfunction of the light-sensitive retinal neurons, photoreceptors, results in irreversible vision loss that adversely affects the quality of the patients' life. More than 146 mutated genes have been identified as causal for inherited photoreceptor diseases^1^ with additional genes constantly being identified (RetNet: https://sph.uth.edu/retnet/disease.htm, provided in the public domain; Retinogenetics: http://retinogenetics.org/, provided in the public domain). This multiplicity in genetic mutations results in photoreceptor death mediated through numerous cell death mechanisms.^1–3^ One key regulator of neuronal function and survival in brain and retinal tissue is the family of myocyte enhancer factor 2 (MEF2) transcription factors.^4–11^ *Mef2c* gene expression is diminished in the *Rpe65*^−^*^/^*^−^ mouse model of Leber's congenital amaurosis (LCA)^12^ and in rd1 mice, a model for photoreceptor degeneration with a homozygous mutation in *Pde6b*.^13^ Moreover, genetic mutations of *Mef2* transcription factors are known to contribute to other forms of human disease. Two recent studies implicated MEF2D in photoreceptor survival with *Mef2d* null mice displaying photoreceptor degeneration.^14,15^ In the present study, we use a genetic knockout model consisting of *Mef2d* null mice to elucidate a new molecular pathway that protects photoreceptors in the setting of MEF2 deficiency. We identify a MEF2D-PGC1α transcriptionally activated cascade, dysregulation of which results in apoptotic photoreceptor death. We also devise an improved retinal explant technique that allowed us to collect molecular, histologic, and functional (electroretinogram [ERG]) evidence that MEF2D-PGC1α transcriptional activation protects photoreceptors from cell death. Our findings suggest that therapeutics aimed at activating the MEF2D-PGC1α cascade may prove effective in combating photoreceptor degeneration in retinal diseases.

## Materials and Methods

### Mouse Colonies

*Mef2d*^−^*^/^*^−^ mice were generated and maintained on a 129Sv/C57BL6 mixed background.^16^ Central nervous system (CNS)-conditional *Mef2c knockout* mice were developed by our group.^17^ Briefly, we crossed mice expressing the nestin-Cre transgene (n-Cre)^18^ with mice carrying the floxed *Mef2c* allele^19^ to obtain the n-Cre^+^ *Mef2c^loxp/loxp^* conditional mice. All animals were housed at our Institute's animal facility. All procedures were performed in accordance with Institutional and National Institutes of Health-approved Guidelines for Animal Research and in compliance with the ARVO Statement for the Use of Animals in Ophthalmic and Vision Research.

### Immunohistochemistry and Measurement of Outer Nuclear Layer (ONL) Thickness

Immunolabeling for MEF2D antibody was performed on retinal cryosections, with details in Supplementary Material. ONL thickness was measured in hematoxylin and eosin (H&E) stained retinal sections as previously described and detailed in Supplementary Material.

### Terminal Deoxynucleotidyl Transferase (TdT) dUTP Nick-End Labeling (TUNEL) Assay

TUNEL assay was performed as per manufacturer's instructions for evaluation of apoptosis. Image analysis details are provided in the Supplementary Material.

### Immunoblotting and Reverse Transcription-Quantitative Polymerase Chain Reaction (RT-qPCR)

Gene and protein expression analysis was performed using RT-qPCR and Western blotting respectively, with details in Supplementary Material.

### Chromatin Immunoprecipitation (ChIP)

ChIP assays were performed using ChIP-IT EXPRESS assay kit (Active Motif, Carlsbad, CA, USA). Immunoprecipitations were performed using anti-MEF2 antibody (sc-313; Santa Cruz, Dallas, TX, USA). Input and ChIP DNA were analyzed by qPCR using LightCycler 480 q-PCR system (Roche, Indianapolis, IN, USA) (see Supplementary Material).

### Retinal Explant Culture Preparation and AAV Treatment

Retinal explants were cultured as previously described with modifications. Details of this procedure and adeno-associated virus (AAV) treatment are provided in the Supplementary Material.

### Electroporation and Luciferase Reporter Gene Assay in Retinal Explants

Retinal explants were electroporated with MEF2 or PGC1α luciferase reporter and Renilla luciferase control vector, as described previously. Analysis was performed using Dual-Glo luciferase assay kit (Promega, Madison, WI, USA). See Supplementary Material.

### ERG of Retinal Explants

Photoreceptor function was evaluated ex vivo by recording microERGs from acute retinal preparations and retinal explants using multielectrode array (MEA). See Supplementary Material for details.

### Statistical Analysis

Data represent at least three independent experiments, presented as mean ± SEM. For 2-way comparisons, statistical significance was ascertained by Student's *t*-test; *P* < 0.05 was considered significant.

## Results

### Abundant MEF2 Expression in Neural Retina

**Figure 1:**
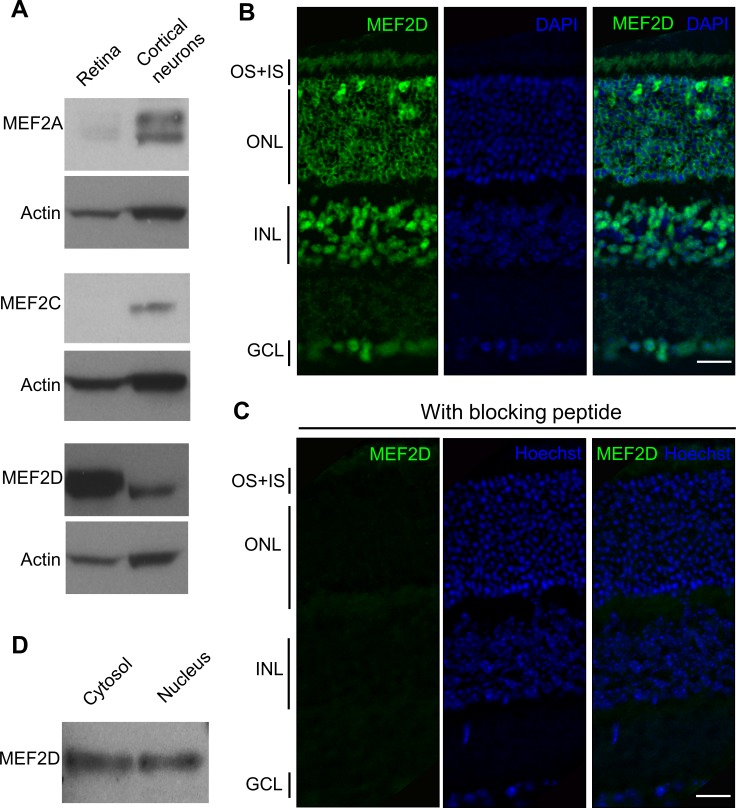
MEF2 expression in mouse retina. (**A**) Comparative protein expression of MEF2A, MEF2C, and MEF2D with actin loading controls in adult rodent retina and adult cerebrocortex by western blot. (**B**) MEF2D immunohistochemistry on mouse retinal sections at P60. (**C**) Preincubation with MEF2D blocking peptide. (**D**) Immunoblot of MEF2D protein in cytosol and nucleus of P60 retinal cell lysates. OS, outer segments; GCL, ganglion cell layer. *Scale bar*: 20 μm.

Previous work had profiled transcripts in mouse retina using an unbiased and comprehensive serial analysis of gene expression (SAGE).^20^ Notably, the number of SAGE tags suggested that MEF2D is the most abundant form of MEF2 transcription factor expressed in the retina. To further validate this finding, we performed western blots to determine the expression levels of the various MEF2 family members in the retina compared to cerebrocortical neurons. We found that MEF2D was the predominant isoform in the retina (Fig. 1A). Next, we examined MEF2D expression by immunohistochemistry in adult mouse retina at 2 months of age. Using multiple antibodies, we observed MEF2D expression in the ONL, inner nuclear layer (INL), and ganglion cell layer (Fig. 1B). MEF2D immunoreactivity was seen in nuclei as well as in nonnuclear compartments, including the cytoplasm of cell bodies and the inner plexiform layer (IPL). Preincubation with the cognate blocking peptide completely ablated the MEF2D immunoreactivity, confirming its specificity (Fig. 1C). The intracellular localization of MEF2D in retina was further investigated by western blot analysis. After subcellular fractionation of retinas, cytosolic and nuclear-enriched fractions were immunoassayed. MEF2D was detected in the cytosol and the nucleus (Fig. 1D), consistent with the immunohistochemical findings above. This conclusion was also confirmed by two recent studies.^14,15^

### Absence of MEF2 Causes Photoreceptor Cell Death

**Figure 2:**
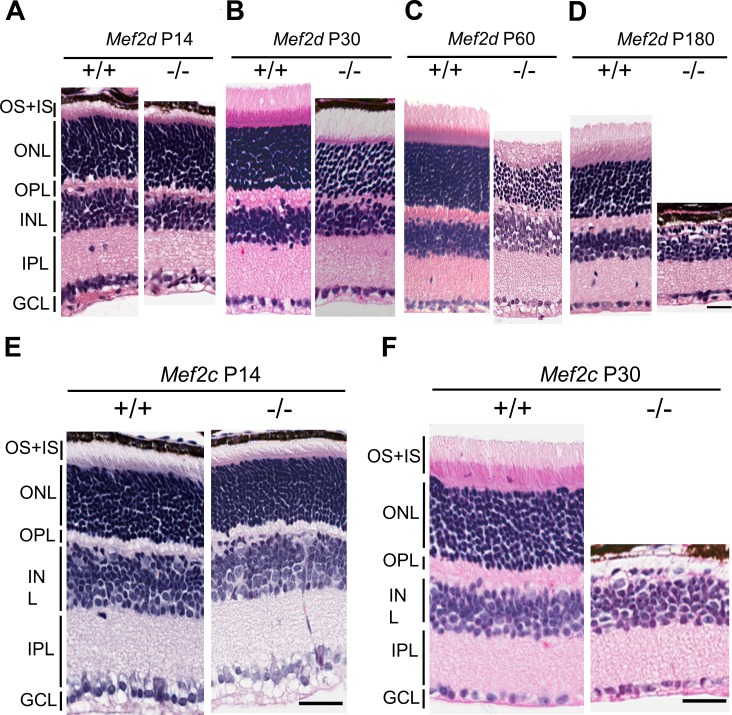
Progressive thinning of ONL in *Mef2d*^−^*^/^*^−^ and *Mef2c*^−^*^/^*^−^ mice. **(A**–**D**) H&E staining of *Mef2d^+/+^* and *Mef2d*^−^*^/^*^−^ retinas at P14 (**A**), P30 (**B**), P60 (**C**), and P180 (**D**). (**E, F**) Representative images of H&E stained retinal sections of *Mef2c^+/+^* and *Mef2c*^−^*^/^*^−^ retinas at P14 (**E**) showing normal structural phenotype, and at P30 (**F**) showing dramatic loss of photoreceptors in *Mef2c*^−^*^/^*^−^ with only a single row of photoreceptors remaining. *Scale bars*: 20 μm.

We next studied morphologic changes in *Mef2d*^−^*^/^*^−^ retina by H&E staining at different ages. There was no obvious morphologic difference between wild-type (WT) *Mef2d*^+/+^ and *Mef2d*^−^*^/^*^−^ retinas at postnatal day (P)14, a time when retinal development is essentially complete^21^ (Fig. 2A). In contrast, by P30 we found a slight reduction in the number of rows at the ONL (Fig. 2B). At P60, we observed a severe reduction in the ONL thickness (Fig. 2C), and by P180, only a single layer of photoreceptors remained in *Mef2d*^−^*^/^*^−^ retinas (Fig. 2D). Using *Mef2c* CNS-specific knockout mice generated in our laboratory,^17^ we found, similar to *Mef2d*^−^*^/^*^−^, that *Mef2c*^−^*^/^*^−^ retinas showed no discernible morphologic difference at P14 compared to WT on H&E staining, but subsequently photoreceptors degenerated rapidly and were almost completely lost by P30 (Figs. 2E, 2F).

**Figure 3:**
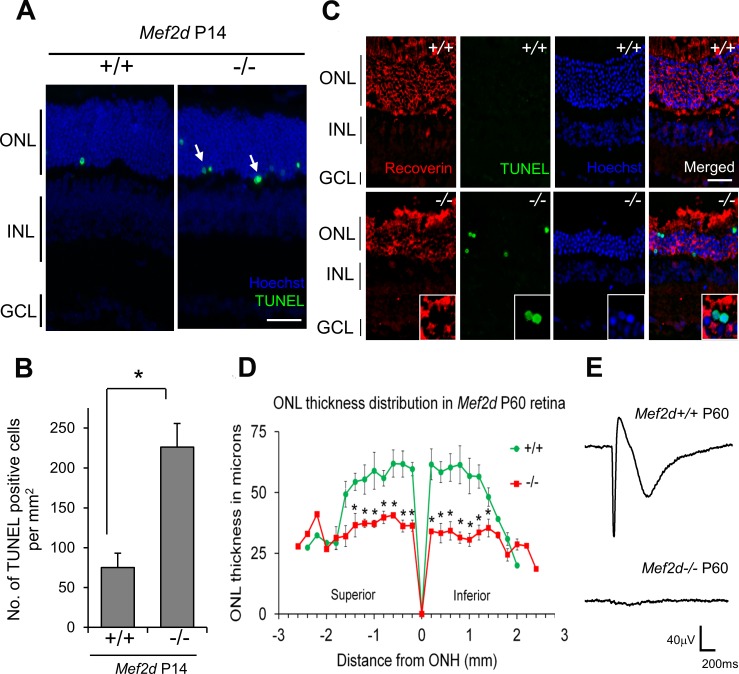
Progressive loss of photoreceptors in *Mef2d*^−^*^/^*^−^ mice. (**A**) Representative photomicrographs of TUNEL stained (*green*) photoreceptors in P14 *Mef2d^+/+^* and *Mef2d*^−^*^/^*^−^ retinal sections. (**B**) Quantification of TUNEL positive cells in P14 retina. Values are mean + SEM (*n* = 3, *P < 0.02). (**C**) Representative images of *Mef2d^+/+^* and *Mef2d*^−^*^/^*^−^ retinas at P60 stained with the photoreceptor marker Recoverin (*red*) and TUNEL (*green*) for apoptotic cell death. (**D**) ONL thickness distribution of *Mef2d^+/+^* and *Mef2d*^−^*^/^*^−^ retinas at P60. Values are mean ± SEM (*n* = 3, *P < 0.05). WT values are similar to those we previously reported.^52^ **(E**) Representative ERG traces recorded using MEA from P60 *Mef2d^+/+^* and *Mef2d*^−^*^/^*^−^ retinas. The a-wave is the initial downward deflection of the trace. *Scale bars*: 20 μm.

Additionally, despite the absence of any gross morphologic difference in the retinas of *Mef2d*^−^*^/^*^−^ and WT mice at P14, we did find a significant increase in the number of TUNEL-positive cells with condensed morphology in the ONL region of *Mef2d*^−^*^/^*^−^ retina (Figs. 3A, 3B), consistent with the notion that photoreceptor degeneration had already begun by this age (note in mouse, 97% of photoreceptors are rods^22^). At P60, we observed increased TUNEL-positive photoreceptors, as demonstrated by Recoverin staining, in the ONL of *Mef2d*^−^*^/^*^−^ mice compared to control WT at P60 (Fig. 3C), indicating ongoing photoreceptor loss. We and others have previously shown that disruption of MEF2 activity results in apoptotic cell death.^10^ Our data in conjunction with prior work therefore suggest that an apoptotic form of cell death may have occurred. To quantify this developmental loss of photoreceptors, we measured ONL thickness at 200-μm intervals starting at the optic nerve head (ONH) and extending toward the superior and inferior ora serrata. The mean ONL thickness at P60 was reduced by approximately 33% in the superior retina and 39% in the inferior retina of *Mef2d*^−^*^/^*^−^ mice compared to WT (Fig. 3D).

We also assessed photoreceptor function ex vivo at P60 by recording ERGs with a MEA. As expected, photoreceptor loss in *Mef2d*^−^*^/^*^−^ mice resulted in the absence of light responses (the “a-wave”) in the ERG (Fig. 3E). Taken together, these findings indicate that *Mef2d*^−^*^/^*^−^ photoreceptors degenerate in an age-dependent manner.

### MEF2D Regulates PGC1α Transcription

**Figure 4:**
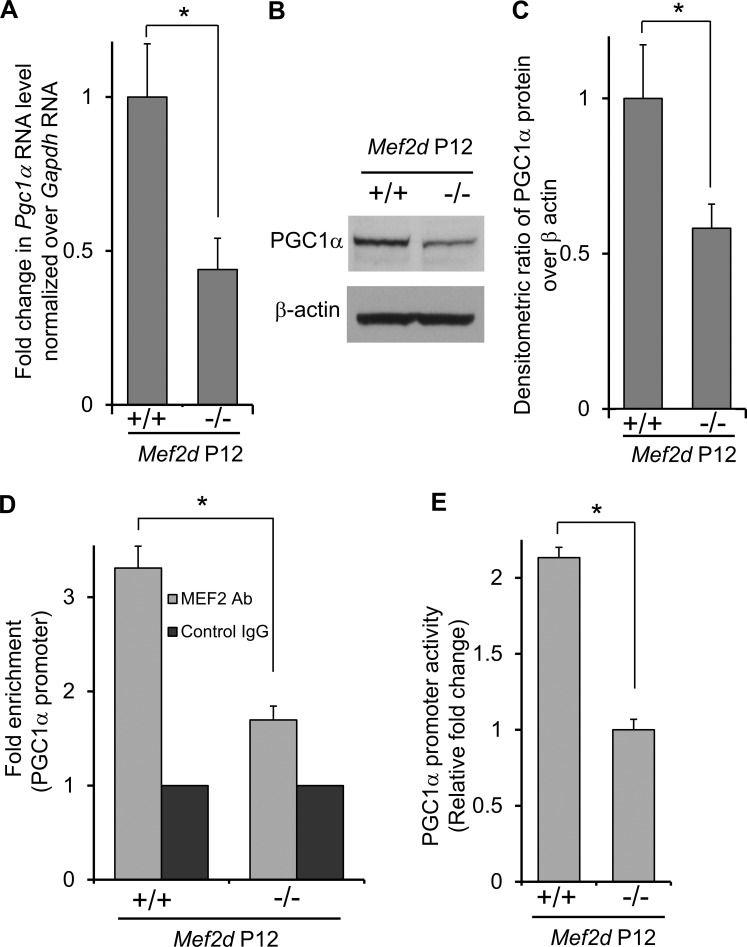
MEF2D promotes PGC1α transcription. (**A**) RT-qPCR expression of PGC1α displays a 50% decrease in *Mef2d*^−^*^/^*^−^ retinas at P12. Values are mean + SEM (*n* = 4, *P < 0.01). (**B**) Western blot representing PGC1α protein levels are lower in *Mef2d*^−^*^/^*^−^ compared to *Mef2d^+/+^* in P12 retinas with actin as loading control. (**C**) PGC1α protein quantification by densitometry of immunoblots (*n* = 5, *P < 0.05). (**D**) ChIP analysis of MEF2D binding to PGC1α promoter in mouse retina. Data represent mean + SEM (*n* = 3, *P < 0.005). (**E**) Luciferase assay shows increased PGC1α promoter activity in *Mef2d^+/+^* compared to *Mef2d*^−^*^/^*^−^ retina normalized to Renilla luciferase. Values are mean + SEM (*n* = 4, *P < 0.002).

We next sought to determine which MEF2 transcriptional targets might contribute to the loss of photoreceptors. Previously, we had performed gene expression profiling on human neural/photoreceptor progenitor cells expressing a constitutively active form of MEF2 in order to identify biological processes controlled by the transcription factor.^23^ Prominent among the MEF2 target genes was peroxisome proliferator-activated receptor γ coactivator 1α (PGC1α), a transcriptional coactivator that induces gene networks controlling mitochondrial biogenesis and antioxidant production.^24–28^ Interestingly, several neurodegenerative disorders, including retinal degenerative diseases as well as Alzheimer's disease and Parkinson's disease, exhibit mitochondrial deficits.^26^ PGC1α is important for maintaining mitochondrial function and is highly expressed in tissues with high-energy demands, including retina.^29,30^ Notably, *Pgc1*α^−^*^/^*^−^ mice exhibit photoreceptor degeneration upon light exposure.^29^ We reasoned therefore that PGC1α might represent a key MEF2 transcriptional target in photoreceptors involved in susceptibility to light damage. To begin to assess functional involvement of MEF2 activity in PGC1α signal transduction, we performed both quantitative (q) reverse transcription PCR analysis and Western blots at developmental stages prior to cell death in *Mef2d*^−^*^/^*^−^ versus WT retinas. Thus, observed differences could be attributed to dysregulation of MEF2-governed transcriptional cascades. We found decreased PGC1α mRNA and protein expression at P12 in *Mef2d*^−^*^/^*^−^ retinas relative to WT control (Figs. 4A–C).

To determine whether MEF2 directly binds to the PGC1α promoter in vivo in retina, we performed ChIP of MEF2 on the PGC1α promoter in P12 mouse retinas. We found a 2-fold increase in MEF2 bound to PGC1α promoter in *Mef2d^+/+^* mice compared to *Mef2d*^−^*^/^*^−^ (Fig. 4D), supporting the view that MEF2 controls PGC1α expression in retina. Further, to obtain direct evidence that MEF2 regulates PGC1α expression in retina, we performed a reporter gene luciferase assay in P1 retinas electroporated with a PGC1α reporter construct. After 4 days in culture, we found elevated promoter activity in the WT compared to *Mef2d*^−^*^/^*^−^ retina, consistent with the notion that MEF2 drives PGC1α expression (Fig. 4E).

### Improved Retinal Explant Culture Method

**Figure 5:**
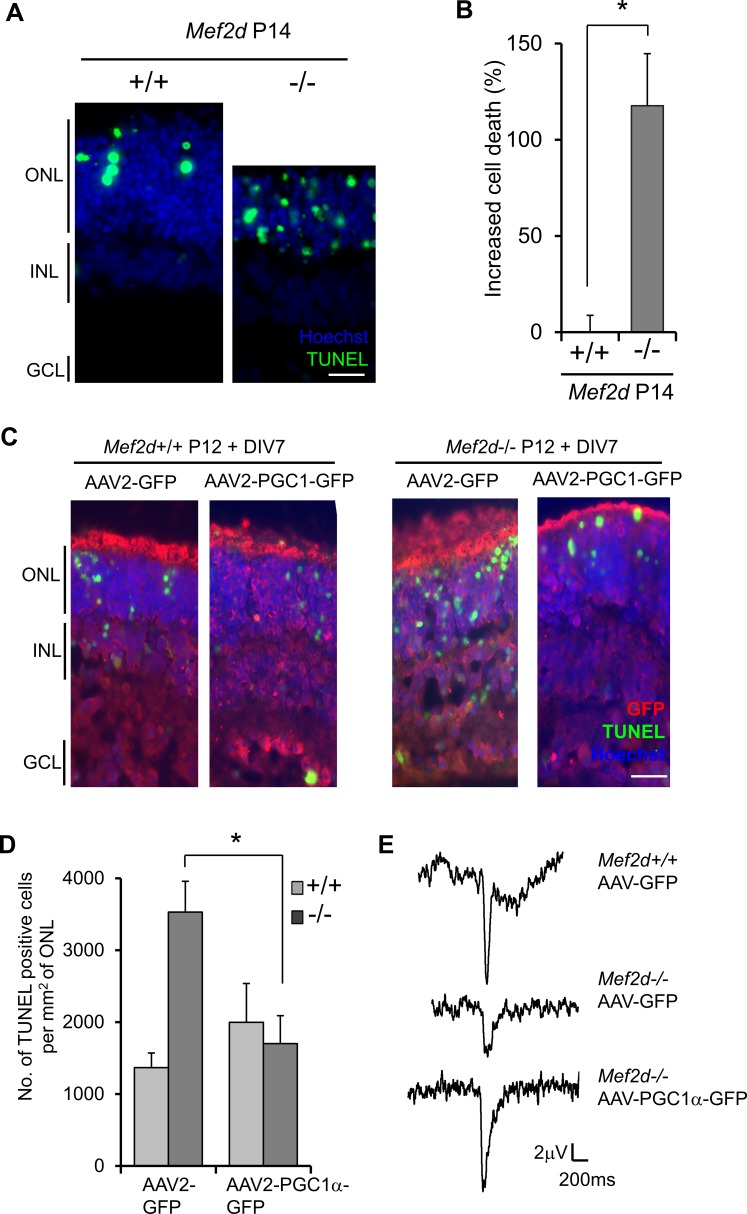
AAV2-mediated repletion of PGC1α rescues photoreceptors in P12 *Mef2d*^−^*^/^*^−^ retinal explants. (**A**) Representative images of TUNEL staining (*green*) on retinal explants cultured in the dark from P12 *Mef2d^+/+^* and *Mef2d*^−^*^/^*^−^ mice after 7 days in vitro (DIV). *Scale bar*: 20 μm. (**B**) Quantification of TUNEL-stained P12+DIV7 explants showed that *Mef2d*^−^*^/^*^−^ photoreceptors manifested significantly more apoptosis above basal level of WT (*n* = 3, *P < 0.05). (**C**) Immunofluorescent images of TUNEL stained WT and *Mef2d*^−^*^/^*^−^ P12+DIV7 retinal explant sections. *Scale bar*: 20 μm. (**D**) Quantification of TUNEL-positive photoreceptor cells in *Mef2d*^−^*^/^*^−^ explants transduced with AAV2-PGC1α compared to empty vector AAV2 (*n* = 4, *P < 0.04). (**E**) Representative ex vivo ERG traces from AAV2 transduced *Mef2d^+/+^* and *Mef2d*^−^*^/^*^−^ P12+DIV5.

Our findings suggest that the MEF2-PGC1α pathway might play an antiapoptotic role in photoreceptors and therefore could represent a novel therapeutic target. To test if augmentation of the MEF2-PGC1α pathway can mitigate photoreceptor loss in degenerative diseases, we employed ex vivo retinal explants because genetic manipulations, such as gene transfer for gain-of-function analysis with genes of interest, can be performed quickly and efficiently in this model. We initially sought to produce a more robust explant system. We modified and improved prior protocols to preserve photoreceptor form and function in WT explants by culturing the retina in a “ganglion-cell-layer-down/photoreceptor-cell-layer-up” orientation and in total darkness, harvesting retinas from dark-adapted P12 animals. This represents an age when retinal development is complete, and photoreceptor cell death has not yet occurred in the *Mef2d*^−^*^/^*^−^ retina. To investigate whether morphology was preserved in WT retinas ex vivo, we assessed nuclear (Hoechst)-stained retinal explant sections isolated after 7 days in culture. The laminar structure in the explants was well maintained (Fig. 5A).

Next, to assess if our ex vivo system recapitulates the degenerative features of *Mef2d*^−^*^/^*^−^ retinas observed in vivo, we performed TUNEL staining on explants from *Mef2d^+/+^* and *Mef2d*^−^*^/^*^−^ mice cultured for 7 days. In accordance with our in vivo data, *Mef2d*^−^*^/^*^−^ explants underwent rapid degeneration, with twice as many apoptotic cells as WT (Figs. 5A, 5B).

### PGC1α Repletion Provides Neuroprotection in *Mef2d^–/–^* Retinal Explants

Having developed and characterized this improved explant model, we then utilized the preparation to investigate the potential antiapoptotic role of the MEF2-PGC1α pathway in retinal photoreceptor degeneration. To determine whether PGC1α can rescue *Mef2d*^−^*^/^*^−^ photoreceptors from cell death, we used a green fluorescent protein (GFP)-labeled AAV2 construct to express PGC1α. We transduced P12 WT and *Mef2d*^−^*^/^*^−^ retinas with AAV2-PGC1α-GFP and cultured them for 7 days. Immunocytochemistry with anti-GFP antibody was consistent with widespread viral transduction (Fig. 5C). To determine the extent of photoreceptor apoptotic cell death, we examined explant sections for TUNEL-positive cells in the ONL. We found a significant decrease in the number of apoptotic photoreceptors after forced PGC1α expression, indicating neuroprotection (Figs. 5C, 5D). Since GFP expression is in the cytoplasm and TUNEL fluorescence in the nucleus, the two signals do not colocalize even if the same cell is labeled.

To evaluate photoreceptor rescue functionally, we recorded ERGs using our MEA platform. Forced expression of PGC1α rescued the photoreceptor a-wave light responses in *Mef2d*^−^*^/^*^−^ explants (Fig. 5E). Taken together, these results show that the MEF2-PGC1α cascade can be therapeutically targeted to improve photoreceptor survival in the retina of *Mef2d*^−^*^/^*^−^ mice.

## Discussion

MEF2 transcription factors are prominent regulators of neurogenesis and neuronal survival in the CNS,^7,10,11,31^ but their role in photoreceptors is only recently emerging.^12–15^ Here, we show retinal photoreceptors express MEF2 transcription factor isoforms similar to many other CNS neurons. Photoreceptors express both MEF2C and MEF2D, with MEF2D apparently the predominant form. MEF2D expression begins during development and continues into adulthood. Our studies confirm that MEF2 is required for photoreceptor survival,^14,15^ and we describe for the first time that lack of MEF2 results in progressive photoreceptor degeneration in a PGC1α-dependent fashion. Interestingly, many of the phototransduction genes downregulated in *Mef2d*^−^*^/^*^−^ retinas are also mutated in human retinal diseases that result in photoreceptor degeneration, including retinitis pigmentosa (RP) and cone-rod dystrophy.^14^ Moreover, a decrease in MEF2 has been observed in other genetic mouse models of RP and LCA, suggesting the potential role of MEF2 transcription factors in human homologues of the disease.^12,13^

Elucidating pathophysiological mechanisms in retinal disease is key to devising efficient therapeutic strategies to delay or prevent cell death.^1^ However, with multiple genes implicated in photoreceptor diseases,^32^ targeting each gene separately would be impractical. Intriguingly, photoreceptors in different diseases encounter diverse cellular stresses that converge onto common cell death mechanisms.^1,33,34^ One model proposes that mitochondrial pathways integrate and control proapoptotic signaling in response to various cellular stresses.^1^ This argues for identification of a central regulator of prosurvival and antiapoptotic pathways as a direct, universal and cost-effective therapeutic approach. Accordingly, in the present study we identify the MEF2D-PGC1α transcriptional pathway as a candidate for therapeutic intervention in photoreceptor diseases, particularly since loss of MEF2D activity results in decreased PGC1α expression.

PGC1α is a transcriptional co-activator regulating mitochondrial bioenergetics and oxidative metabolism and is highly expressed in cells with large energetic demands including photoreceptors.^25,26^ Further, lack of PGC1α renders photoreceptors susceptible to light damage.^29^ Given its crucial role in mitochondrial function, PGC1α has been identified as a therapeutic target for several neurodegenerative diseases.^35–37^ Supporting this hypothesis, we provide evidence for transcriptional regulatory influence of MEF2D on PGC1α expression and for a neuroprotective role of PGC1α in photoreceptors. Using adeno-associated viral transduction, our data demonstrate that repletion of PGC1α in *Mef2d*^−^*^/^*^−^ photoreceptors prolongs neuronal survival and preserves the functional light response. Since mitochondrial dysfunction is implicated in the etiology of a number of retinal diseases, including diabetic retinopathy, glaucoma, and AMD,^38^ this pathway might also serve as a therapeutic target in these retinal diseases. While we found that the MEF2D-PGC1α pathway contributes to photoreceptor survival, our findings of course do not rule out the potential importance of other survival pathways.

During the course of our studies, to facilitate our evaluation of the role of the MEF2D-PGC1α pathway in photoreceptor survival, we developed an improved retinal explant method. Culturing isolated retina can be difficult due to the fragility of photoreceptors and the high metabolism of retina.^39^ Several protocols have been developed in an attempt to address these issues to maintain cell viability in various species at different stages, both with and without intact retinal pigment epithelium (RPE).^40–45^ Unfortunately, RPE-photoreceptor co-culture restricts access to the photoreceptor layer; however, removing the RPE reduces the ability of photoreceptors to reverse the consequences of light adaptation and to survive for prolonged periods.^46^ To circumvent these issues, we cultured RPE-free retinal explants in total darkness after harvesting from dark-adapted mice. Recently improved explants methods have been introduced,^47–49^ but, in contrast to those techniques, our approach cultured and harvested the explants in the dark, which improved their viability under our conditions. Using our procedure and placing the retinas photoreceptor-side-up, we were able to maintain photoreceptor viability, structure, and function for at least 7 days. Under our conditions, culture in the dark allowed photoreceptors to retain their light responsiveness. In one other study, ERG responses in mouse retinal explants have been reported to be preserved, but that study co-cultured the retina with RPE cells.^50^ In our preparation, we observed an a-wave on the microERG, corresponding to functional photoreceptor light responses. While there was no subsequent b-wave in the ERG in this preparation, corresponding to second-order neuronal responses, this was not critical as our studies were focused on preservation of photoreceptor function. We speculate that the early development of the retina in our preparation or our microERG technique may have favored resolution of the a-wave over the b-wave. In any event, the lack of b-wave can be viewed as an experimental advantage because this technique allowed us to dissect out the effects of the MEF2D-PGC1α pathway on photoreceptors (assessed via a-wave responses) as opposed to second-order cells (b-wave dominant).

Interestingly, the Cepko laboratory recently reported that PGC1α overexpression accelerates cone photoreceptor cell death in an RP mouse model.^51^ This result would seem to differ from our observations, as we found a neuroprotective effect of PGC1α in our rod-dominant photoreceptor mouse model. This apparent discrepancy may possibly be explained by the fact the prior study tested the effect of PGC1α in a mouse model after all of the rods had already died and only the cones remained. Importantly, we expressed PGC1α in our retinal model before rod photoreceptor cell death had begun. Moreover, in the context of MEF2D deficiency, PGC1α is underexpressed compared to WT. Hence, in our paradigm, correction of this deficiency, rather than overt overexpression of PGC1α, results in rescue of photoreceptors.

In summary, we identify the MEF2D-PGC1α transcriptional cascade as a neuroprotective pathway for retinal photoreceptor disease, particularly in the context of MEF2D deficiency. Employing our improved explant culture method, we show that AAV-mediated PGC1α gene therapy ameliorates photoreceptor cell death and preserves photoreceptor light responsiveness. In conjunction with prior studies, our results highlight the notion that the level of PGC1α may represent a bell curve with respect to photoreceptor survival; overt overexpression may contribute to photoreceptor cell death,^51^ whereas maintenance of the PGC1α pathway may be required for survival, as observed here.

## Supplementary Material

Supplement 1
