# Supplementary material for: Molecular Pathway to Protection From Age-Dependent Photoreceptor Degeneration in Mef2 Deficiency
Source: Invest Ophthalmol Vis Sci. 2017 Jul;58(9):3741–9. doi: 10.1167/iovs.17-21767 (PMC5525556; doi:10.1167/iovs.17-21767)
Supplement: Supplement 1 [file iovs-58-07-64_s01.pdf]

Supplementary Material

Molecular Pathway to Protection from Age-Dependent Photoreceptor Degeneration in *Mef2* Deficiency

Saumya Nagar, Dorit Trudler, Scott R. McKercher, Juan Piña-Crespo, Nobuki Nakanishi, Shu-ichi Okamoto, and Stuart A. Lipton

CONTENTS

Supplementary Experimental Procedures

Supplementary References

Supplementary Experimental Procedures

Immunohistochemistry

Retinas were enucleated from mice and fixed in 4% PFA overnight and cryopreserved in 30% sucrose in PBS. Cryosections were then blocked with 10% normal serum, 3% BSA, 0.3% Triton X-100 in PBS at RT for 1 h. Tissues were incubated overnight with a primary anti-MEF2D antibody (1:200, Santa Cruz, sc-271153; 1: 2000, BD biosciences, #610774; 1:250, Abcam, ab32845; data shown for Santa Cruz antibody); subsequent incubation was performed in an appropriate Alexa Fluor 488/594 conjugated secondary antibody diluted 1:250 for 1 hour at RT. Sections were counterstained with Hoechst 33342 from Thermo Scientific.

For the blocking peptide experiments, antibody (Santa Cruz, sc-271153) was preincubated with a five-fold (by weight) excess of the antibody-specific blocking

peptide in 500 µL PBS for 2 hours at RT and 4°C. The preincubated antibody-peptide solution was used for immunochemistry following the same protocol as described above.

Immunoblotting

Retinas were extracted in PBS, homogenized in T-PER Tissue Protein Extraction Reagent (Thermo Scientific) containing protease inhibitor cocktail (Thermo Scientific) and centrifuged to obtain protein samples in solution. Protein concentration was estimated using BCA reagent (Thermo Scientific). Equal amounts of protein samples separated by gel electrophoresis, and transferred to nitrocellulose/PVDF membranes. The following antibodies were used to assess and quantify protein expression: MEF2C (1:2000, Aviva, ARP37342_T100), MEF2A (1:1000; Aviva, ARP37186_T100), MEF2D (1:2000; BD biosciences), and PGC1α (1:2000; Millipore, ST1202).

Measurement of Outer Nuclear Layer (ONL) Thickness

Mice were enucleated and the orientation of the eye was marked with animal tattoo ink on the cornea (Ketchum Manufacturing, Inc., Ontario, Canada) in the superior region. After fixation with 4% paraformaldehyde, eyes were embedded in paraffin and 5 µm thick sections were cut through the optic disc of each eyeball for staining with hematoxylin and eosin (H&E). ONL thickness was measured in retinal sections within the optic nerve head region, starting at the optic nerve head (ONH) and extending along the vertical meridian toward the superior and inferior ora serrata at 200 µm intervals using Aperio Image Scope software. The mean ONL thickness was calculated for the entire retinal section using 6 fields-of-view per section [1] and averaged across three sections within the optic nerve head.

Terminal Deoxynucleotidyl Transferase (TdT) dUTP Nick-End Labeling (TUNEL) Assay

In Situ Cell Death Detection Kit (Fluorescein; Roche) was used to determine apoptotic cell death of photoreceptors. For assessment of apoptosis, retinal sections within the optic nerve region were subjected to TUNEL assay according to the manufacturer's protocol. Images were taken throughout the length of the ONL. TUNEL-positive cells were counted per mm^2 of ONL and averaged across all sections.

Chromatin Immunoprecipitation

Chromatin immunoprecipitation (ChIP) assay was performed using the ChIP-IT EXPRESS assay kit as per the manufacturer's protocol (Active Motif, Carlsbad, CA). Briefly, wild-type control and *Mef2d*$^{-/-}$ retinas were collected at P12 and homogenized. Cells were incubated in 1% formaldehyde at room temperature for 10 min, washed with PBS and lysed, followed by centrifugation at 2400*g*. The nuclear pellet was resuspended in shearing buffer and sonicated with 20 pulses of 15 s duration to shear DNA into 500-1000 bp. Ten percent of this mixture was stored for 'input DNA' analysis. An equal amount of the protein/DNA complex mixture was then incubated with magnetic beads and rabbit IgG or anti-MEF2 antibody (C-21; Santa Cruz Biotechnology, sc-313) at 4°C overnight. Immunoprecipitated DNA was then eluted from the magnetic beads and cross-linking was reversed. Input and ChIP DNA were analyzed using qPCR. The PGC1α promoter region was amplified using forward primer 5′-TGGCTGTAATGAGAACTTCAATCA-3′ and reverse primer 5′-GTAGCTCCATCAAATCAGCAGTT-3′ for determination of MEF2 binding. For quantitative ChIP, the PCR was performed using SYBR green on the Lightcycler 480 q-PCR system (Roche). Levels of enrichment (n-fold) were calculated using the comparative cycle threshold method (Ct method).

Reverse Transcription Quantitative Polymerase Reaction (RT-qPCR)

Total RNA was extracted from retinal samples using the *mir*Vana RNA extraction kit (Ambion). RNA concentration was determined using a Nanodrop Spectrophotometer (Thermo Scientific),

and equal amounts of RNA were used to set up a RT-qPCR reaction using the EXPRESS One-Step Superscript qRT-PCR Kit (Invitrogen) in a Lightcycler 480 qPCR instrument (Roche). All reactions were performed in triplicates and normalized to GAPDH expression.

Retinal Explant Cultures

For explant preparation, P12 wild-type control and *Mef2d*$^{-/-}$ retinas were dissected with the lens intact under sterile conditions in the dark under a red safelight with Wratten filter #2. Retinas were then carefully transferred with the photoreceptor side up onto polycarbonate membranes floating on culture media (1:1 ratio of DMEM:F12, 10% FBS, 100 U/mL penicillin, 100 µg/mL streptomycin, 0.29 mg/mL L-glutamine, 5 µg/mL insulin). A drop of media was placed on each retina to allow air exchange without tissue drying, and the retinas were cultured in total darkness for 7 days at 37°C in a 5% CO_2/balance air incubator.

AAV Treatment of Explants

Modified AAV2 with 7m8 capsid (a kind gift of Dr. John Flannery at the University of California, Berkeley) engineered to express GFP and human PGC1α were prepared at the academic viral core facility of the Children's Hospital of Philadelphia (CHOP), Pennsylvania. This AAV2 construct had been used previously to infect photoreceptors preferentially [2]. Explants were incubated in total darkness overnight with AAV2 (6x10^12 vg/mL), and culture media was replaced with fresh culture media containing 10% serum. After 7 days of culture in total darkness, explants were fixed and cryosectioned. Anti-GFP antibody was used to visualize GFP expression using Alexa Fluor 594 conjugated secondary antibody (red); this allowed us to enhance the GFP signal that was localized in the cytoplasm. TUNEL staining was performed on sections taken every 300 µm, and TUNEL-positive cells were counted per mm^2 of ONL throughout its length.

Electroporation of Retinal Explants for Luciferase Reporter Gene Assay

Dark-adapted retinas, dissected in serum-free explant culture media under a red safelight with a Wratten #2 filter, were placed in an electroporation chamber (model BTX453 Microslide chamber, Harvard Apparatus) with the photoreceptors oriented towards the negative electrode. The chamber contained 80 µL of 0.5 µg/µL PGC1α firefly luciferase reporter construct along with 0.1 µg/µL renilla luciferase control vector. Using an electroporator (model ECM 830), five 30 V square pulses of 50 ms duration every 950 ms were applied. This enabled selective transfer of the plasmids to photoreceptors [3, 4]. Electroporated retinas were then recovered in serum-free media for 10 min and placed in 10% FBS-containing media for an additional 10 min. Retinas, with lens side down, were then transferred onto polycarbonate membranes in culture media and cultured at 37°C, 5% CO_2 in total darkness. Electroporated explants were harvested after four days of culture and analyzed using a Dual-Glo luciferase assay kit (Promega) as per the manufacturer's instructions.

Electroretinograms (ERGs)

MicroERGs were recorded using a multielectrode array (MEA, 60 electrodes (30 µm diameter/200 µm inter-electrode distance) arrayed as an 8×8 square grid) to analyze photoreceptor function *ex vivo* from acute retinal preparations (*n* = 3 per genotype) and retinal explants (*n* = 3 per condition). Eyes from dark-adapted mice were enucleated and quickly dissected under dim red light (Wratten filter #2) in carbogen bubbled Ames' Medium (Sigma, A1420) buffered with sodium bicarbonate ($NaHCO_3$), Ames' media at 34°C. Retinas were carefully extracted from the eyecup, flattened, and mounted on a carrier filter paper a 2-mm hole in the center. The filter paper with the retina was then placed on the MEA with the ganglion cell layer in contact with the electrodes. In the case of retinal explants, explants from P12 retinas cultured in the dark for 5 days were used without filter paper. A weighted mesh ring was used to hold the sample in place. Retinas were perfused with Ames' Medium (Sigma, A1420) at 20 mL/min flow rate for 20 min and maintained at 34°C. A Uniblitz shutter (Vincent Associates)

connected to the microscope light source and driven by a computer controlled stimulus generator (STG2004, MultiChannel Systems) was used to elicit light responses of 20-1000 ms. Recorded traces from each electrode were extracted and converted into axon binary file format (.ABF) using MC Data Tool software (Multi Channel Systems) and analyzed using pClamp10 software (Molecular Devices). Traces were filtered at 300-500 Hz using a lowpass filter (Bessel, 8-pole). Responses peak amplitude and duration were measure using pClamp10.

Supplementary References

1. Rezaie T, McKercher SR, Kosaka K, et al. Protective effect of carnosic acid, a pro-electrophilic compound, in models of oxidative stress and light-induced retinal degeneration. *Invest Ophthalmol Vis Sci.* 2012;53:7847-7854.

2. Dalkara D, Byrne LC, Klimczak RR, et al. In vivo-directed evolution of a new adeno-associated virus for therapeutic outer retinal gene delivery from the vitreous. *Sci Transl Med.* 2013;5:189ra176.

3. Donovan SL, Dyer MA. Preparation and square wave electroporation of retinal explant cultures. *Nat Protoc.* 2006;1:2710-2718.

4. Montana CL, Myers CA, Corbo JC. Quantifying the activity of cis-regulatory elements in the mouse retina by explant electroporation. *J Vis Exp.* 2011;52:pii 2821.
